# Case 4/2019 - 26-Year-Old Man with Congenital Chagas Disease and Heart Transplantation

**DOI:** 10.5935/abc.20190162

**Published:** 2019-08

**Authors:** Henrique Trombini Pinesi, Tânia Mara Varejão Strabelli, Vera Demarchi Aiello

**Affiliations:** Instituto do Coração (InCor) - Hospital das Clínicas da Faculdade de Medicina da Universidade de São Paulo (HC-FMUSP), São Paulo, SP - Brazil

**Keywords:** Heart Defects, Congenital, Chagas Cardiomyopathy, Heart Transplantation, Heart Failure, Diagnosis Imaging

A 26-year-old man with cardiopathy due to congenital Chagas’ disease was submitted to heart transplantation for heart failure; amastigote forms of *Trypanosoma cruzi* were found in an endomyocardial biopsy in the third month after transplantation.

The patient had been diagnosed with Chagas’ disease due to transplacental transmission and was followed at Hospital das Clínicas of Faculdade de Medicina of Universidade de São Paulo (FMUSP) until the age of 2 years, when he was discharged from the follow-up. He was diagnosed with cardiopathy due to Chagas’ disease at age 20.

He sought emergency medical care on July 23, 2016, at age 25, for dyspnea on moderate exertion and paroxysmal nocturnal dyspnea and edema for three weeks.

The physical examination disclosed blood pressure of 118/98 mmHg, heart rate of 87 bpm, respiratory rate of 28 breaths/min, oxygen saturation of 99% and pulmonary auscultation showed decreased vesicular murmur at the bases. Cardiovascular examination disclosed increased jugular venous pressure, thin pulses, cardiac stroke deviated 2 cm beyond the nipple line, 2 digital pulps, arrhythmic heart sounds, normal heart sounds with the presence of a third heart sound and mitral systolic murmur. Abdominal examination revealed painful hepatomegaly, with the liver palpated 6 cm from the right costal border and ++ /4 edema in the lower limbs.

The electrocardiogram showed sinus rhythm, heart rate of 107 bpm, PR interval of 187 ms, QRS duration of 146 ms, left atrial overload, right bundle-branch block and anterosuperior left bundle-branch block and probable left ventricular overload (Figure 1).

**Figure 1:**
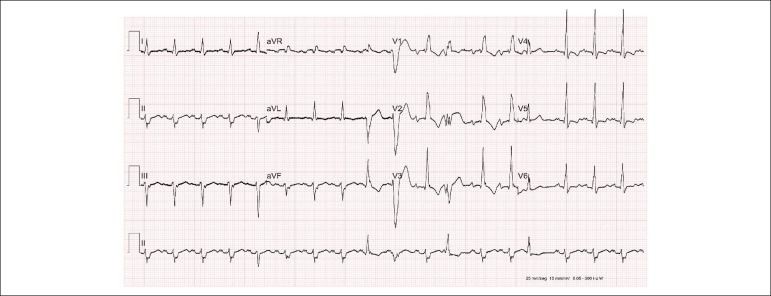
Electrocardiogram. Sinus rhythm. Left atrial enlargement, right bundle branch block, left anterior hemiblock, premature ventricular contraction, premature atrial contraction.

The posteroanterior chest x-ray disclosed veil-like opacification of the both hemithoraces, compatible with pleural effusion, increased pulmonary hila with signs of pulmonary congestion and cephalization of the pulmonary vasculature network and ++++ / 4 global cardiomegaly (Figure 2).

**Figure 2:**
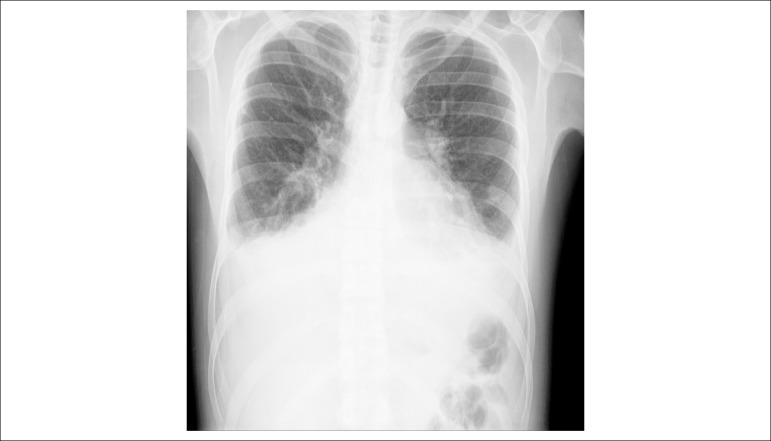
PA chest x-ray: veil-like opacification of pulmonary bases (pleural effusion), global cardiomegaly.

The patient was re-hospitalized on July 29, after two episodes of syncope that occurred on the day before hospitalization.

The physical examination disclosed a patient in regular overall status, hydrated, eupneic, acyanotic, conscious and oriented. Blood pressure was 90x60 mmHg, heart rate was 88 bpm, oxygen saturation was 97%; pulmonary auscultation was normal; cardiac auscultation disclosed an irregular heart rhythm, with no heart murmurs or accessory sounds; the abdominal examination showed no visceromegaly, no lower-limb edema, and no calf stiffness.

Laboratory tests (July 29, 2016) showed: red blood cells: 5200000/mm³, hemoglobin 15.6g/dL, hematocrit of 47%, leukocytes 14,570/mm³ (74% of neutrophils), platelets 157,000/mm³, potassium 3.8 mEq/L, sodium 137 mEq/L, BNP 1,128 pg/mL, ALT 54 U/L, AST 42 U/L, gamma-GT 99 U/L. Urinalysis was normal.

The chest x-ray (July 29, 2016) disclosed clear pulmonary fields and cardiomegaly at the expense of the right ventricle (Figures 3A and B).

**Figure 3:**
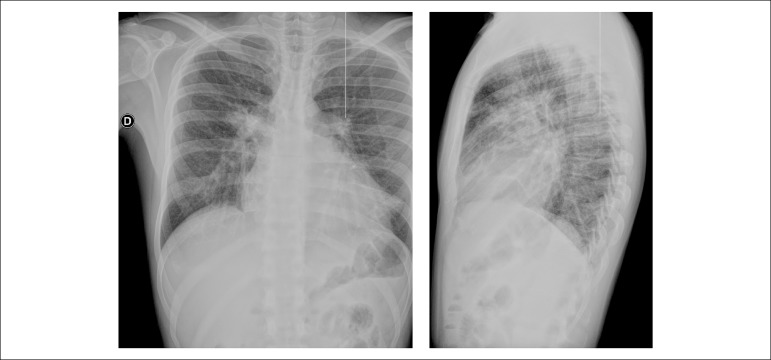
X-rays: global cardiomegaly, larger increase of the right ventricle.

The transthoracic echocardiogram (July 29, 2016) showed: left atrium, 52 mm; right ventricle, 43x32 mm; septum, 8 mm; posterior wall, 8 mm; left ventricle 66x60 mm; left ventricular ejection fraction, 20%; pulmonary artery systolic pressure, 39 mmHg. There was a marked increase in left atrium (volume measured by Simpson's rule was estimated at 61 mL/m^2^, normal value < 34 mL/m^2^); moderate enlargement of the left ventricle and right atrium; slight enlargement of the right ventricle. Systolic function was decreased due to diffuse hypokinesia. The right ventricle showed mild hypokinesia. There was also mild to moderate mitral regurgitation, as well as mild to moderate tricuspid regurgitation.

The heart MRI (August 1, 2016) showed marked right ventricular dilatation with an ejection fraction of 17%, left ventricle with diffuse hypokinesia, with late mesocardial enhancement in the basal segment of the septum and transmural enhancement in the basal, middle and apical segments of the anterior, lateral and inferior walls, and less than 50% in the basal inferosseptal segment. The ejection fraction of this ventricle was also 17% (Figure 4).

**Figure 4:**
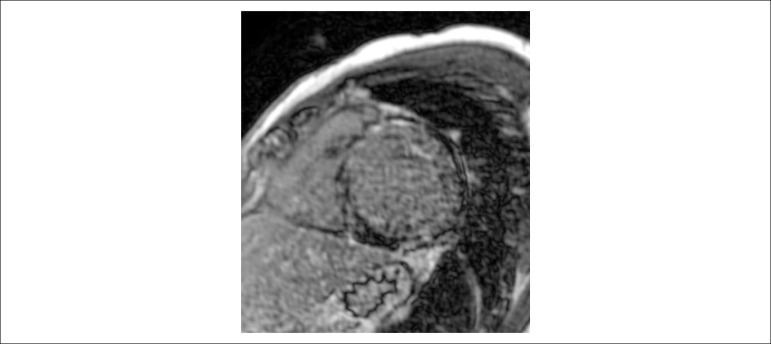
Magnetic resonance image: transmural, mid-wall and subepicardial late gadolinium enhancement not involving subendocardium.

In the electrophysiological study (August 4, 2016) there was no onset of atrial or ventricular arrhythmias after the extra stimuli.

Right catheterization (August 23, 2016) showed mean right atrial pressure of 11 mmHg, right ventricular end-systolic and diastolic pressures of 45/29 mmHg, and pulmonary capillary pressure of 30 mmHg. The pulmonary vascular resistance was 3.6 Wood Units (normal: 0.25 to 1.6 Wood U) and the cardiac index was 2.1L / min / m². After the use of 10 µg/kg/min of dobutamine, pulmonary vascular resistance decreased to 1.6 Wood U.

The patient was placed on a transplant waiting list with a priority status, as vasoactive drug weaning was not achieved due to hypotension. Orthotopic heart transplantation was performed with no complications on December 6, 2016. The donor was positive for cytomegalovirus.

The anatomopathological analysis of the para-aortic lymph nodes revealed reactive lymphadenitis with no granulomas.

The post-transplantation electrocardiogram (December 09, 2016) showed low voltage in the frontal plane and end-conduction disorder (Figure 5).

**Figure 5:**
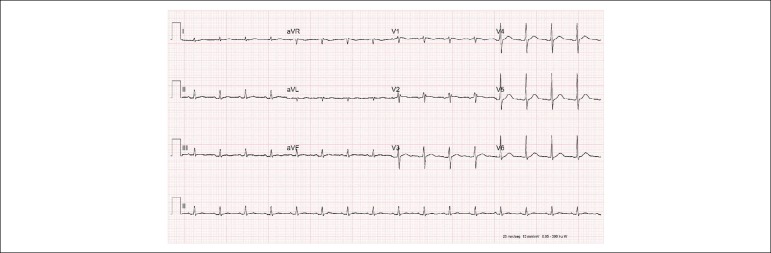
Post-transplantation electrocardiogram: low frontal plane voltage and end-conduction disorder.

The biopsy on December 16, 2016 showed focal mild-degree fiber aggression; focal mild histiocytic proliferation and mild focal lymphocytic infiltrate; there was moderate diffuse edema. Compatible with acute grade 1R cellular rejection (low grade mild rejection). The search for antibody-mediated rejection by immunohistochemical reaction to complement C4d fraction was negative.

The serology was negative for cytomegalovirus; however, molecular biology screening for the parasite was positive since the end of December and viral load reached 47417 U/mL in February 2017, when the patient received ganciclovir for 21 days.

The echocardiogram performed in February 2017 was normal, except for an increase in the left atrium, whereas the one performed in March showed all measures within the normal range.

In a biopsy carried out in March 2017, moderate and focal fiber aggression, moderate diffuse proliferation, moderate focal lymphocytic infiltrate and mild diffuse edema were observed. Amastigote nests were observed inside the myocytes, with protozoal myocarditis. This biopsy was suggestive of Chagas' disease reactivation, with moderate mononuclear myocarditis. Kinetoplasts were observed in the parasites and the immunohistochemistry was positive for *Trypanosoma cruzi* antigens. Benznidazole was then prescribed.

A biopsy carried out in September 2017 disclosed acute grade 2R cellular rejection (moderate rejection, intermediate grade). Both histological and immunohistological analysis were negative for *Trypanosoma cruzi*.

The serology for Chagas' disease was negative in November 2017.

At an outpatient consultation on May 3 2019, the patient was asymptomatic and the physical examination was normal.

## Clinical aspects

Chagas' disease was first described by Brazilian physician and scientist Carlos Chagas in 1909.^1^ This multifaceted disease is caused by the protozoan *Trypanosoma cruzi*, which can be transmitted in different ways. Vector transmission through hematophagous insects is the most classic one, although it has declined in importance in recent years with measures to control the vector population.^2^ Transfusion transmission, as well as vector transmission, have shown a drastic reduction in the last decades, and no cases have been reported in Brazil for years.^3^ In contrast, there was an increase in reports of oral transmission. This form of contagion was little known but gained importance, with several descriptions of micro-epidemic events in the country, especially related to the consumption of açai berry. The development of the acute phase of the disease is more common in the oral transmission.^4^ In the context of controlling the main forms of contagion, vertical transmission has become relevant.

The World Health Organization (WHO) estimates there are approximately 8 million individuals infected with Chagas disease worldwide, with an annual mortality of 10,000 people due to disease complications.^5^ Most of these cases are found in Latin America, and Brazil is the country with the majority of infected individuals (approximately 4.6 million individuals). The decrease in transmission was accompanied by a reduction in mortality, estimated in 2007 to be 2.78 deaths/year for every 10,000 inhabitants.^6^ Another change in the disease epidemiology in recent years has been the increase in cases in non-endemic regions, such as the USA and Europe, which has contributed to the increased attention given by the international scientific community to Chagas’ disease.^5^

The pathophysiology of Chagas' disease is multifactorial, depending on several characteristics of both the host and the parasite.^7^ It is known that the inflammatory response triggered by the parasite plays a crucial role in this pathophysiology.^8^ This hypothesis is supported by low tissue parasitism and low parasitemia in the chronic phases of the disease. More recent studies have identified an autoimmune response triggered by the cross-reaction between parasite antigens and host proteins, such as troponin.^9^ Diagnosis is attained by serological tests in the vast majority of cases, with the direct investigation of the parasitic agent being reserved for acute phases or reactivations, situations in which the parasitemia may be higher.^7^

Congenital Chagas’ disease is a separate group. It occurs when there is vertical transmission, that is, during pregnancy. In Brazil, Martins-Melo et al.^10^ demonstrated that the mean prevalence of infected pregnant women is 1.7%, with a mean percentage of congenital transmission of 1.7%. Extrapolating these data to the population based on the 2010 census, Brazil would have approximately 34,629 infected pregnant women, with an incidence of 587 children born with congenital Chagas per year.^10^ Due to these numbers, the WHO recommended in 2018 increased attention to cases of congenital Chagas through maternal-fetal transmission, not only in Brazil, but in all countries with endemic disease.

In a study carried out in Argentina in 2014, Fabbro et al.^11^ demonstrated that children of pregnant women who received treatment with antitrypanosomal drugs during their lives have a much lower chance of developing congenital Chagas’ disease than children of women who have not been treated.^11^ Thus, in addition to indications of etiological treatment included in the I Latin American Guideline for Chagas' disease in 2011, it is recommended that women of childbearing age also receive antitrypanosomal drugs. In Brazil, the available drug is benznidazole, which should be used at a dose of 5mg/kg/day divided into 2 or 3 doses a day for 60 days.^7^ It is worth noting that benznidazole use is contraindicated during pregnancy, due to the risk of teratogenicity found in animal studies.^12^

The clinical picture of congenital Chagas disease is extremely variable and non-specific, being similar to several other infections seen in the neonatal period, such as toxoplasmosis, rubella, HIV and syphilis. The main symptoms are preterm birth, intrauterine growth restriction, neuropsychomotor development deficit, low Apgar score, respiratory distress syndrome, jaundice, and hepatosplenomegaly. These symptoms may appear days or even weeks after birth. Mortality is approximately 5% and is usually related to more severe manifestations, such as meningitis and myocarditis.^13^ The diagnosis, in addition to the clinical picture, is based on the direct screening for the parasite up to 6 months of age and on the serological tests after 9 months, due to the presence of circulating antibodies from the mother. All cases should be treated with antitrypanosomal drugs as soon as the diagnosis is confirmed.^14^ The earlier treatment is implemented, the lower the incidence of side effects and the higher the cure rate, which is 100% when implemented in the first year of life.^12^

Chagas' disease reactivation can affect any individual with the chronic forms of the disease, especially when they are submitted to immunosuppression. In this context, the most important conditions are HIV coinfection and/or organ transplantation.^14^ Among patients with Chagas cardiopathy submitted to heart transplantation, the incidence varies between 21 and 45% depending on the studied series.^15^ Review studies show that mortality is low when appropriate treatment is implemented and, therefore, diagnosis is a crucial part of a favorable outcome.^16^

The diagnosis of reactivation in the transplanted patient is based on the clinical picture and the routine screening for the parasite in endomyocardial biopsies, because often there are no symptoms, or the symptoms are non-specific. Symptoms may be cardiac, such as congestive and low-output symptoms in cases of myocarditis or changes in the electrocardiogram, such as cardiac rhythm disturbances or new blocks. The most frequent extracardiac symptoms are fever and skin lesions. Thus, a high degree of suspicion is necessary for diagnosis to be attained. When the endomyocardial biopsy is altered, the main differential diagnosis is acute cellular rejection, since there is lymphocyte/monocyte infiltration in both cases. The difference is that nests of *Trypanosoma cruzi* amastigotes may be present in the Chagas’ disease reactivation biopsy, observed by hematoxylin-eosin staining or by immunohistochemistry analysis.^15^

When the reactivation diagnosis is made, the recommended treatment is benznidazole use, at the previously mentioned doses. It should be emphasized that this treatment does not result in the cure of the chronic infection, and the patient is subject to recurrent reactivations. As a consequence of this risk, it is recommended that the immunosuppression in these patients be as little as possible, aiming at an adequate balance between the risk of reactivation and rejection.^15^

Azathioprine use should be preferred instead of mycophenolate in the Chagasic population, since it is associated with a lower reactivation rate, without worsening of other outcomes.^17^ Thus, despite the drastic decrease in the disease transmission in the country, Chagas' cardiopathy continues to be very frequent in Brazil, both due to the number of patients with the chronic forms of the disease and to other forms of transmission that were not previously very relevant, such as vertical transmission. The care of pregnant women and the treatment of those infected at reproductive age should be improved. The active screening for infection in the children of infected mothers is essential, aiming at establishing the earliest possible antitrypanosomal treatment in infected children, thus achieving the cure and reducing the potential number of patients with the chronic forms of the disease. **(Dr. Henrique Trombini Pinesi)**

**Diagnostic hypothesis:** Chagas disease reactivation in transplanted heart. **(Dr. Henrique Trombini Pinesi)**

## Infectious aspects

The laboratory monitoring of Chagas' disease reactivation after cardiac transplantation is recommended. Due to the difficulty of attaining a clinical diagnosis of Chagas’ disease reactivation, with the exception of skin lesions, the laboratory monitoring of transplanted patients is recommended.^15^ For this purpose, peripheral blood samples should be collected for direct detection of the parasite in the buffy coat, which increases the likelihood of its finding, and for parasite screening by molecular biology. This method seems to be more sensitive, detecting the increase of the parasitic load before the onset of clinical and/or histopathological manifestations. It can be qualitative or quantitative. Monthly or quarterly monitoring is recommended in the first year after cardiac transplantation, when the level of immunosuppression is higher and after the treatment of rejection episodes. **(Prof. Dr. Tânia Mara Varejão Strabelli)**

## Anatomopathological report

**Figure 6:**
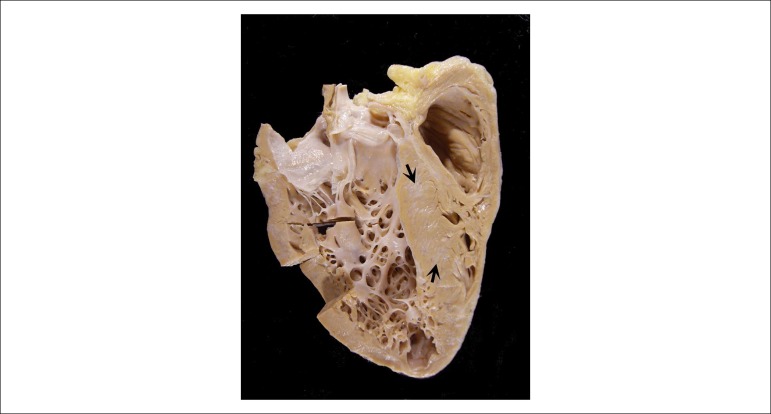
Longitudinal section of the explanted heart, showing chamber dilatation and areas of fibrosis in the ventricular septum (arrows).

**Figure 7:**
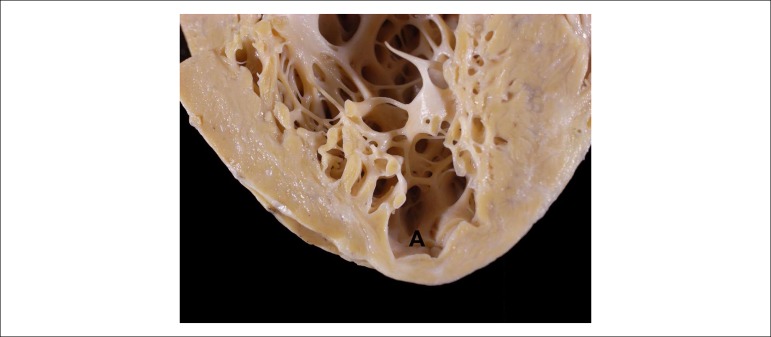
Detail of the left ventricular tip showing the typical lesion of chronic Chagasic cardiopathy, characterized by myocardial tapering with aneurysm formation (A).

The explanted heart weighed 332g. It had a globose shape and the external surface was covered by smooth serosa, with small white and prominent nodules being noticed, focally, in the trajectory of the coronary vessels. The opening showed dilatation of all chambers (Figure 6), predominantly of the ventricles. The endocardial surface was smooth, showing no thrombi. The left atrial endocardium was quite thick. The left ventricular tip showed a dilated lesion measuring 1.2 cm in diameter, where the wall was tapered and partially replaced by whitish tissue (Figure 7). The epicardial coronary arteries showed no macroscopic alterations, as well as the atrioventricular and arterial valves. Histological analysis showed chronic mononuclear myocarditis and diffuse fibrosis, of which intensity varied from one region to another. We did not find parasites in the histological sections of the explanted heart.

**Figure 8:**
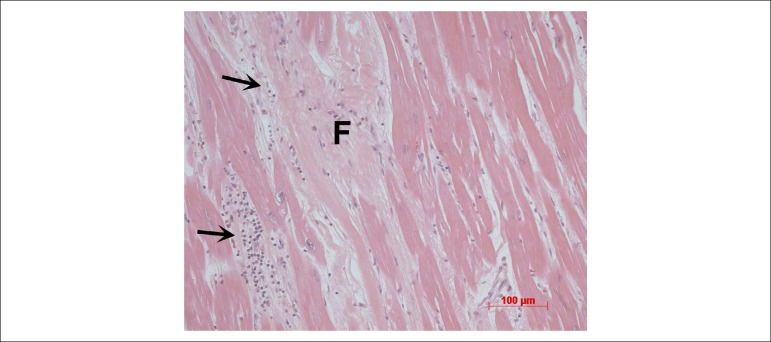
Myocardial photomicrography of the explanted heart, showing extensive fibrosis (F) and active myocarditis foci (arrow). Hematoxylin-eosin staining, magnification = 20X.

The post-transplantation endomyocardial biopsy carried out in March 2017 showed good tissue representativeness, with a moderate inflammatory process and several cardiomyocyte aggression foci (Figure 8).

**Figure 9:**
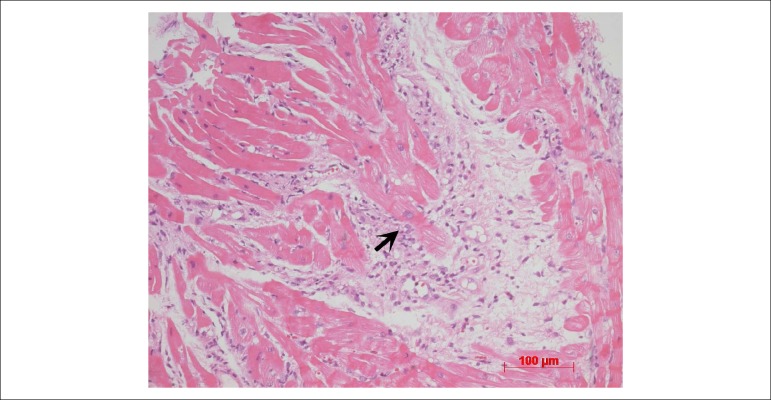
Endomyocardial biopsy photomicrography for post-transplant rejection control. Diffuse inflammatory picture with cardiomyocyte aggression foci can be observed (arrow). Hematoxylin-eosin staining, magnification = 20 X.

**Figure 10:**
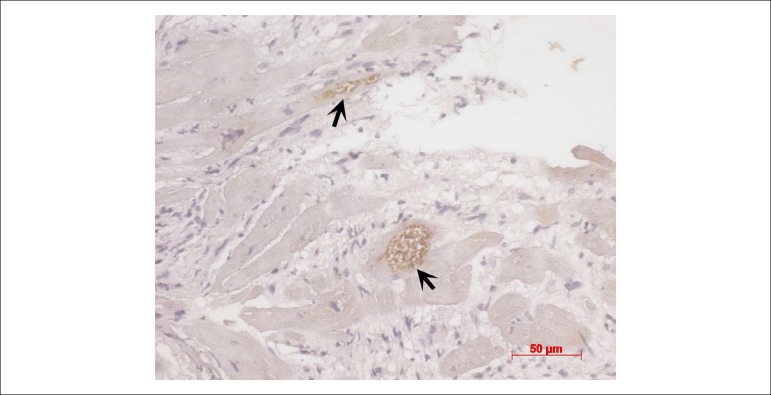
Photomicrograph of the endomyocardial biopsy histological section, submitted to immunohistochemical reaction for T. cruzi parasites. Nests of parasite amastigotes (arrows) are shown in brown. Harris hematoxylin counterstaining, magnification = 40X.

As this was a patient with Chagas’ disease, an immunohistochemical reaction was carried out for *Trypanosoma cruzi* parasites, which were positive in pseudocysts containing amastigotes (Figure 9). New sections of the same block stained with hematoxylin-eosin also showed the presence of pseudocysts (or nests) containing several amastigote forms (Figure 10). **(Dr. Vera Demarchi Aiello)**

## Anatomopathological diagnoses

**Explanted heart**: Chronic myocarditis with diffuse fibrosis, compatible with cardiac involvement in chronic Chagasic cardiopathy.

**Post-transplantation endomyocardial biopsy**: Chagas' disease reactivation, with moderate mononuclear myocarditis and presence of several parasite nests. **(Dr. Vera Demarchi Aiello)**

## Comments

This case shows a young patient with chronic Chagasic cardiopathy who had clinical manifestations around the age of 20 years, after a diagnosis of congenital Chagas' disease. There was no clear history of childhood disease treatment. The explanted heart showed a typical picture of Chagas’ chronic heart disease.

Regarding the post-transplant endomyocardial biopsy findings, the presence of inflammation with more than one focus of cardiomyocyte aggression is, at first, compatible with the diagnosis of acute grade 2R cellular rejection.^18^ However, as this is the case of a patient with Chagas' disease as the primary cardiopathy, one must carry out a more detailed investigation of parasites, since the histological picture of acute 2R cellular rejection is identical to that of disease reactivation. The investigation was then carried out by immunohistochemistry and then in more detailed sections of the biopsy block, we concluded it was a reactivation of Chagas' disease in the transplanted heart, which allowed the appropriate treatment to be implemented.

It is known that the rate of reactivation depends on the implemented immunosuppressive treatment, as previously described. The work of Vidal et al.^19^ also showed that the first episode of reactivation occurred at a median of 6.6 months post-transplantation. Therefore, the routine evaluation of endomyocardial biopsies in Chagasic patients with heart transplantation should include, whenever there is an R2 or higher grade acute cellular rejection, a very detailed evaluation of the histological sections for possible detection of parasites. **(Dr. Vera Demarchi Aiello)**
